# FOSL2-driven SASP in endometrial stroma promotes the inflammation of endometriosis

**DOI:** 10.1038/s41514-026-00447-w

**Published:** 2026-07-24

**Authors:** Weiwei Shi, Xinyi Tang, Fei Yang, Han Yin, Quan Zhou, Fangyue Sun, Shanbo Ding, Udo Jeschke, Lin Peng

**Affiliations:** 1 Department of Thyroid and Breast Surgery, Affiliated Hospital of North Sichuan Medical College, Nanchong, Sichuan China; 2 Department of Obstetrics and Gynecology, Zhongda Hospital Affiliated to Southeast University, Nanjing, Jiangsu China; 3 Center for Reproductive Medicine and Obstetrics and Gynecology Nanjing Drum Tower Hospital Affiliated Hospital of Medical School, Nanjing University, Nanjing, Jiangsu China; 4 The General Public Hospital of Zhangjiagang, Zhangjiagang, Jiangsu China; 5 University of Augsburg, Faculty of Medicine, Gynecology and Obstetrics, Augsburg, Germany

**Keywords:** Cell biology, Diseases, Immunology, Medical research, Pathogenesis

## Abstract

Endometriosis (EMs) is characterized by chronic pelvic inflammation, but the etiology of this inflammation remains poorly understood. The senescence-associated secretory phenotype (SASP), whereby senescent cells secrete pro-inflammatory cytokines, is a potential mechanism. This study investigates the pro-inflammatory SASP in EMs and its underlying influences. Through molecular assays and single-cell RNA-seq analysis, we found a subgroup of endometrial stromal cells (ESCs) marked by SASP in both eutopic endometrium and endometriotic lesions of EMs patients. The transcription factor FOSL2 was aberrantly overexpressed in this ESC subgroup; its overexpression induced cellular senescence and the secretion of SASP factors, while FOSL2 knockdown reversed these effects. Conditioned medium from ESCs with high FOSL2 expression promoted M2 macrophage polarization and recruitment. Mechanistically, FOSL2 overexpression in ESCs was regulated by the PGE2/cAMP/PKA signaling pathway, and FOSL2 modulated SASP through the activation of NF-κB signaling. In conclusion, the SASP in ESCs, regulated by FOSL2, contributes to chronic pelvic inflammation and immune system disruption in EMs patients. Targeting FOSL2 to reverse the SASP may offer a promising therapeutic strategy for EMs.

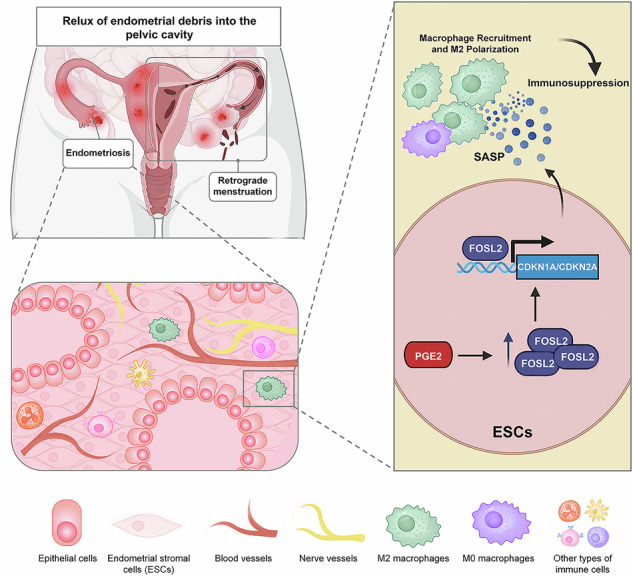

## Introduction

Endometriosis (EMs) is a chronic estrogen-dependent disorder characterized by the presence of endometrial-like tissue outside the uterus, affecting approximately 10% of women of reproductive age worldwide^1^. The most widely accepted theory regarding the pathogenesis of endometriosis is that of the “retrograde menstruation phenomenon”, which suggests that endometrial cells from menstrual blood regurgitate into the pelvic cavity, where they adhere and grow^2^. While the exact mechanisms underlying EMs remain largely unclear, it is well-established as a chronic inflammatory disorder^3^. Significant alterations in immune cell populations, particularly macrophages, as well as inflammatory factors such as interleukin (IL)-1β, IL6, IL8, and C-C motif chemokine ligand 20 (CCL20), have been observed in peritoneal fluid (PF) and endometriotic lesions^4–6^. This inflammatory microenvironment is thought to contribute to pelvic pain, the formation of fibrosis/scar tissue, and even infertility^7^. However, the cause of this inflammatory response remains uncertain.

Cellular senescence is characterized by an irreversible arrest in cell proliferation, accompanied by phenotypic changes, resistance to apoptosis, and the activation of damage-sensing signaling pathways^8^. Senescence can be induced by various intrinsic and extrinsic stimuli, including oxidative stress, mitochondrial dysfunction, oncogenic activation, and exposure to chemotherapeutic agents^9^. Although senescent cells are growth-arrested, they remain metabolically active and secrete a variety of factors collectively referred to as the senescence-associated secretory phenotype (SASP)^10^. The SASP is a dynamic and heterogeneous phenomenon in which senescent cells secrete multiple cytokines, chemokines, growth factors, bioactive lipids, and proteases, many of which exhibit pro-inflammatory properties^11^. Through the SASP, senescent cells interact with various immune cells, contributing to both beneficial outcomes, such as tumor immune surveillance and tissue repair^12^, and detrimental effects, including chronic inflammation and fibrosis^13^. Given that chronic inflammation and fibrosis are hallmark features of endometriosis, it is plausible that the SASP contributes to the development of EMs.

Fos-like antigen 2 (FOSL2/FRA-2), a recently identified member of the Fos family, forms dimers with Jun family members to create activator protein 1 (AP-1) transcription factor complexes^14^. These complexes are involved in the reprogramming of gene expression in response to various stimuli and play critical roles in physiological and pathological processes, including those related to endocrinology, inflammation, and tumor progression^15,16^. FOSL2 has been identified as a key factor in the progression of aggressive cancers, with its elevated expression sustaining pro-inflammatory signaling pathways such as IL6-JAK-STAT3 and IL-17, ultimately leading to immunosuppression within the tumor microenvironment^17^. Recent studies have also implicated FOSL2 in the SASP; for example, Ding and colleagues demonstrated that FOSL2 drives senescence in human liver progenitor-like cells by regulating pro-inflammatory factors such as CCL2, CCL20, and intercellular adhesion molecule-1 (ICAM1)^18^.

In this study, we report for the first time the identification of a distinct subgroup of endometrial stromal cells (ESCs) secreting SASP in EMs. We demonstrate that this SASP-associated ESC subgroup secretes pro-inflammatory factors, which may underlie the chronic pelvic inflammation observed in EMs. Furthermore, we elucidate a mechanism whereby FOSL2 activates the NF-κB pathway to induce the SASP. We also show that the secretome from this ESC subgroup promotes macrophage recruitment and polarizes them toward an M2 phenotype, thereby contributing to the immunosuppressive pelvic microenvironment characteristic of EMs. Our findings underscore the potential of targeting the SASP as a novel therapeutic strategy for EMs.

## Results

### Both eutopic endometrium and lesions of EMs exist in ESCs featured with SASP

**Fig. 1:**
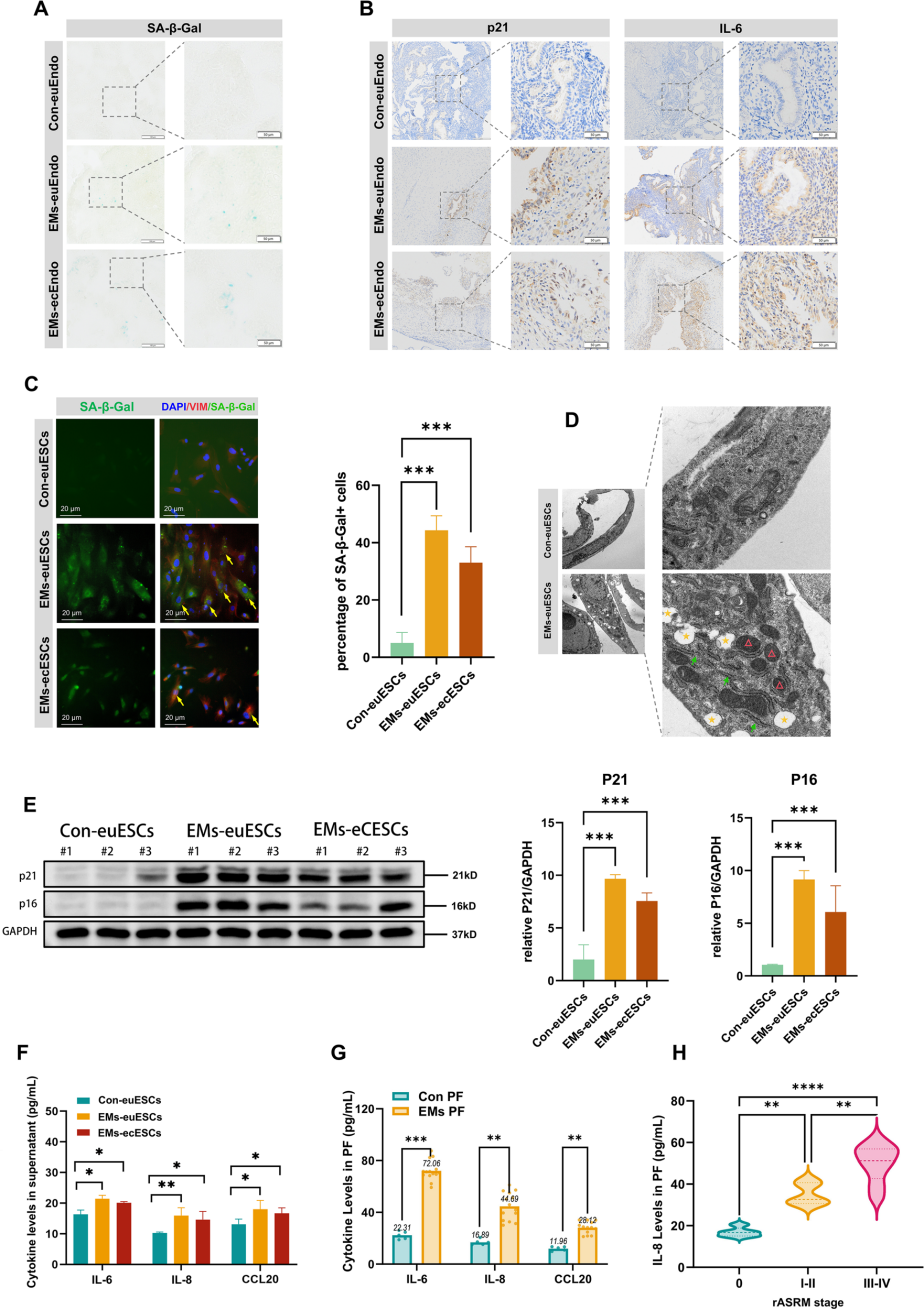
Identification of SASP in EMs. Representative images of (**A**) SA-β-Gal staining, (**B**) p21 and IL6 expression in endometrium from non-EMs controls and EMs patients, and EMs lesions. Arrows highlight cells with positive staining for p21 and IL6 (scale bar, 200 μm and 50 μm). **C** Representative fluorescent images of vimentin (VIM, red fluorescence) and SA-β-Gal (green fluorescence) in ESCs derived from the endometrium of non-EMs controls (Con-euESCs) and EMs patients (EMs-euESCs), and ESCs derived from EMs lesions (EMs-ecESCs) (scale bar, 20 μm). Quantification of the percentage of SA-β-Gal-positive cells was also shown. **D** Transmission electron microscopy (TEM) images of Con-euESCs and EMs-euESCs. Arrows indicate the Golgi apparatus; pentagrams indicate secretory vesicles; triangles indicate lysosomes. **E** The protein levels of p21 and p16 in Con-euESCs, EMs-euESCs and EMs-ecESCs. **F** The concentration of IL6, IL8, and CCL20 in the supernatant of Con-euESCs, EMs-euESCs and EMs-ecESCs. **G** The concentration of CCL20, IL8, and IL6 in PF from non-EMs controls and EMs patients. **H** The concentration of IL-8 in PF of EMs patients at different disease stages. * *P* < 0.05, ** *P* < 0.01, *** *P* < 0.001.

Cellular senescence was confirmed by a combination of SA-β-Gal staining, expression of senescence-associated markers, and transmission electron microscopy (TEM) to assess morphological features such as lysosomal accumulation and secretory vesicle formation. First of all, we performed SA-β-Gal staining on eutopic endometrium from EMs patients and non-EMs controls, as well as on EMs lesions. SA-β-Gal-positive cells were observed in both eutopic endometrium and lesions from EMs patients, while minimal SA-β-Gal-positive cells were detected in eutopic endometrium from non-EMs controls (Fig. 1A). The expression of the typical cell cycle arrest marker p21 and the primary inflammatory cytokine IL6 was also assessed in these tissue groups via immunohistochemistry. Higher protein expression of p21 and IL6 was observed in both eutopic endometrium and lesions from EMs patients, compared to the control eutopic endometrium (Fig. 1B). Notably, SA-β-Gal, p21- and IL6-positive cells were primarily identified as ESCs rather than glandular epithelial cells (Fig. 1A, B). To further validate the presence of SASP in ESCs from EMs, we isolated ESCs and performed immunofluorescence of vimentin (VIM, a marker of ESCs) and SA-β-Gal staining. As shown in Fig. 1C, ESCs derived from eutopic endometrium and lesions of EMs patients (referred to as EMs-euESCs and EMs-ecESCs, respectively) showed clear SA-β-Gal positivity, whereas ESCs from control eutopic endometrium (Con-euESCs) exhibited minimal SA-β-Gal staining. Quantification confirmed that the percentage of SA-β-Gal-positive cells was significantly higher in both EMs-euESCs and EMs-ecESCs compared to Con-euESCs. We then collected ESCs from control and EMs endometrium for TEM examination. In the ESCs of EMs patients, numerous secretory vesicles were observed surrounding the Golgi apparatus, indicating heightened secretory activity. Additionally, abundant lysosomes were present in the ESCs of EM patients, further suggesting that these cells are in a senescent state (Fig. 1D). In line with the senescent phenotype observed, Western blot analysis revealed that the protein levels of the cell cycle markers p21 and p16 were also increased in EMs-euESCs and EMs-ecESCs (Fig. 1E). The mRNA levels of key cell cycle arrest markers p21, p16, and SASP markers CCL20, IL6, and IL8 were significantly elevated in EMs-euESCs and EMs-ecESCs compared to con-euESCs, with the exception of CXCL2 (Supplementary Fig. 1A). The concentrations of pro-inflammatory SASP factors, including IL6, IL8, and CCL20, were also significantly higher in the culture supernatants of EMs-euESCs and EMs-ecESCs compared to those of Con-euESCs (Fig. 1F). Furthermore, these factors were also found to be elevated in the peritoneal fluid (PF) of EMs patients relative to control individuals, with IL-6 exhibiting the most pronounced increase (Fig. 1G). We also found that the levels of IL-8 in the PF significantly increased with the progression of EMs disease stage, suggesting that SASP factors may contribute to the advancement of EMs (Fig. 1H).

In addition, we assessed the proliferative capacity of ESCs across the different groups. The proliferation curves in Supplementary Fig. 1B show that EMs-euESCs and EMs-ecESCs exhibited a slightly slower growth rate compared to Con-euESCs. Moreover, the proportion of EdU-positive cells, which indicates DNA replication activity, was also lower in EMs-euESCs and EMs-ecESCs than in Con-euESCs (Supplementary Fig. 1C). Collectively, these findings confirm the presence of the pro-inflammatory SASP in ESCs of EMs.

### Identification of SASP-associated ESC subgroup based on scRNA-Seq of EMs

**Fig. 2:**
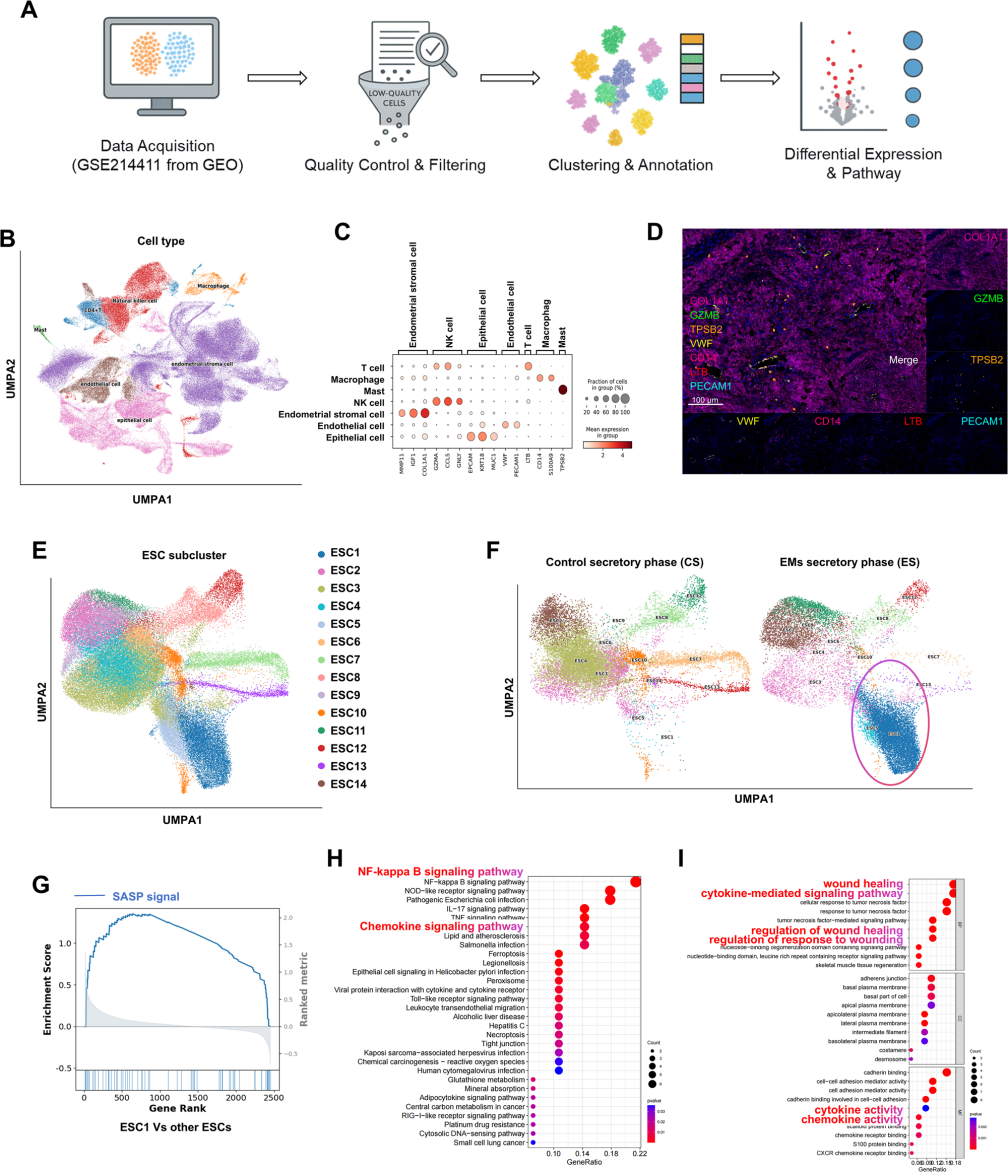
scRNA-Seq analyses of EMs. **A** Schematic workflow for the analysis of public scRNA-seq datasets from eutopic endometrium of control and EMs patients. **B** Representative Uniform Manifold Approximation and Projection (UMAP) plot of the 7 different clusters revealed by Seurat analysis conducted in Python, and cluster identification. **C** Dot plot for common cell-specific markers, such as MMP11, IGF1, COL1A1, GZMA, CCL5, GNLY and S100A9. Cell type classification and the percentage and average expression of the specific cell markers are shown. **D** Representative multiplex immunofluorescence (mIF) images validating the protein expression of key markers used for cell type annotation in human endometrial tissues (Scale bar, 100 µm). **E** UMAP plot of the 13 subgroups of ESCs. **F** UMAP plot of the ESC subcluster of the control secretory phase (CS) and the EMs secretory phase (ES). **G** Enrichment score of SASP signal in ESC1 subgroup. Dot plot of the (**H**) KEGG enrichment analysis and (**I**) GO enrichment analysis for the DEGs of the ESC1 subgroup. Only the most significant DEGs (log_2_FC > 1 and *P*_adj_ < 0.01) were chosen for pathway enrichment analysis. The graph shows the number of genes modulated in every single pathway, the fold enrichment, and the statistical significance.

Since the cells secreting SASP in EMs were predominantly ESCs, and the classical retrograde menstruation theory, along with extensive research indicating that eutopic ESCs are pivotal in the development of EMs^19,20^, we downloaded and re-analyzed the public single-cell RNA sequencing (scRNA-seq) data (GSE214411 from GEO) of eutopic endometrium from EMs patients and non-EMs controls following the workflow outlined in Fig. 2A to deeply explore the SASP-associated ESCs and their characteristics. Following batch correction, cell clustering, and annotation, seven major cell types were identified based on the datasets (Fig. 2B). These cell types, annotated based on previous research^21^, included: ESCs (markers: MMP11, IGF1, COL1A1), natural killer cells (NK cells, markers: GZMA, CCL5, GNLY), epithelial cells (markers: EPCAM, KRT18, MUC1), endothelial cells (markers: VWF, PECAM1), CD4+ T cells (marker: LTB), macrophages (markers: CD14, S100A9), and mast cells (marker: TPSB2). The dot plot in Fig. 2C shows the classification of cell types, along with the percentage and average expression of specific markers. The protein-level expression of key markers was further validated by multiplex immunofluorescence in endometrial tissues (Fig. 2D). The UMAP and the bar plot (Supplementary Fig. 2A, B) display the cell clusters representing the four groups (proliferation or secretory phase endometrium from either non-EMs controls or EMs patients), with the proportional contributions revealing significant heterogeneity between the eutopic endometrium of EMs patients and non-EMs controls. We then further performed subclustering of eutopic ESCs, resulting in the identification of thirteen subclusters (ESC1 to ESC13, Fig. 2E). Notably, the ESC1 subgroup showed a marked elevation in EMs patients, particularly during the secretory phase, in contrast to the control group where the ESC1 subgroup was nearly undetectable (Fig. 2F). Additionally, based on the SASP gene set from the REACTOME database^22^ (R-HSA-2559582, 39 core genes), SASP signaling was activated in the ESC1 subgroup (Fig. 2G). To further characterize this SASP signature, we examined additional well-established markers. Other key pro-inflammatory SASP components, including IL1A, IL1B, and CXCL1, were also highly expressed in the ESC1 subpopulation, while the senescence-associated nuclear integrity marker LMNB1 was downregulated (Supplementary Fig. 2C). To experimentally validate these transcriptomic findings, we performed RT-qPCR on primary cells. This analysis confirmed that IL1A, IL1B, and CXCL1 were significantly upregulated, while LMNB1 was significantly downregulated in ESCs from endometriosis patients (EMs-euESCs and EMs-ecESCs) compared to control ESCs (Supplementary Fig. 2D). KEGG enrichment analysis revealed activation of the NF-κB and chemokine signaling pathways within the ESC1 subgroup (Fig. 2H). Consistently, GO enrichment analysis indicated an upregulation of cytokine and chemokine activities in this subgroup (Fig. 2I). Taken together, the bioinformatics analysis of scRNA-seq datasets reaffirmed the presence of ESCs with a SASP signature in patients with EMs.

### FOSL2 is the key transcription factor regulating SASP in ESCs

**Fig. 3:**
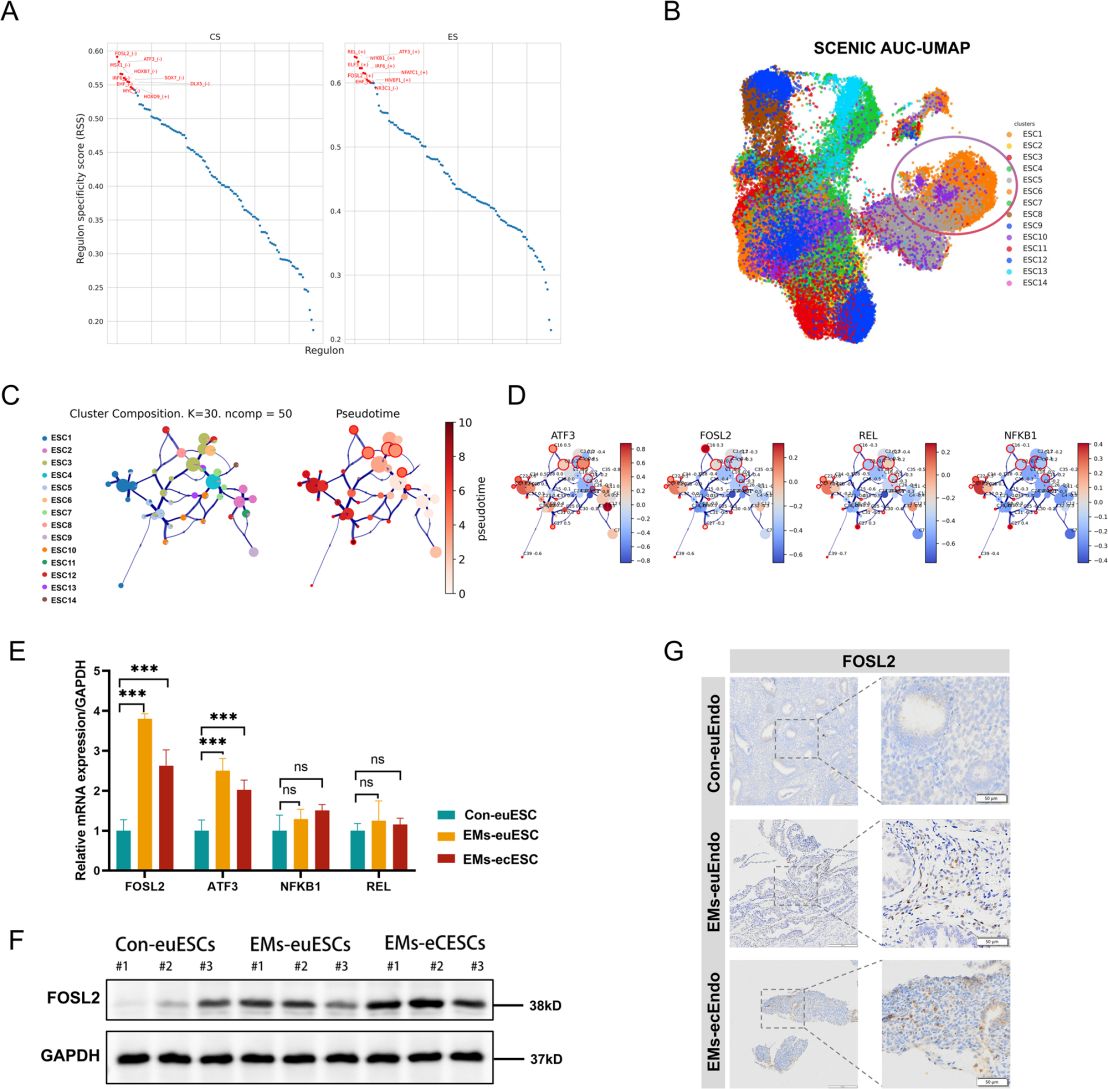
Characterization of subtype-specific TFs in ESCs. **A** Distribution plot of the top 10 differentially expressed TFs of the ESCs from the CS and ES groups. **B** SCENIC AUC-UMAP showing the 14 ESC subclusters. **C** Pseudotime trajectory colored by the pseudotime subcluster of the TFs. **D** Expression of ATF3, FOSL2, REL and NFκB1 along the pseudotime trajectory. **E** The mRNA levels of key TFs FOSL2, ATF3, NFKB1 and REL in Con-euESCs, EMs-euESCs and EMs-ecESCs. **F** The protein expression and corresponding quantification of FOSL2 in Con-euESCs, EMs-euESCs and EMs-ecESCs. **G** Representative immunohistochemical images of FOSL2 expression in eutopic endometrium from non-EMs controls and EMs patients, and EMs lesions. Arrows highlight representative FOSL2-positive cells (scale bar, 200 μm and 50 μm). ** *P* < 0.01, *** *P* < 0.001, ns, no significance.

To investigate the underlying cause of the SASP-associated ESC1 subgroup, we analyzed differentially expressed transcription factors (TFs) in ESCs from EMs and control individuals during the secretory phase. The top 10 differentially expressed TFs for control and EMs ESCs were identified in Fig. 3A. Subsequently, the transcriptional regulatory dynamics across the 14 ESC subclusters were analyzed using SCENI, which revealed heterogeneous TF activity patterns (Fig. 3B). Pseudotime trajectory analysis further positioned the ESC1 subcluster at the terminal end of the differentiation continuum (Fig. 3C). Among the top differentially expressed TFs, only ATF3, FOSL2, REL, and NFKB1 exhibited high expression specifically within the ESC1 subcluster along this trajectory (Fig. 3D). Therefore, we measured the mRNA levels of these TFs, and FOSL2 was identified as the most prominently upregulated TF in both EMS-euESCs and EMS-ecESCs (Fig. 3E). Consistent with this finding, we detected elevated protein levels of FOSL2 in both EMS-euESCs and EMS-ecESCs as compared to Con-euESCs (Fig. 3F). Furthermore, the upregulation of FOSL2 was also observed in the eutopic endometrium and endometriotic lesions from EMs patients compared to the endometrium from non-EMs controls (Fig. 3G).

### FOSL2 regulates SASP based on NF-κB activity in ESCs

**Fig. 4:**
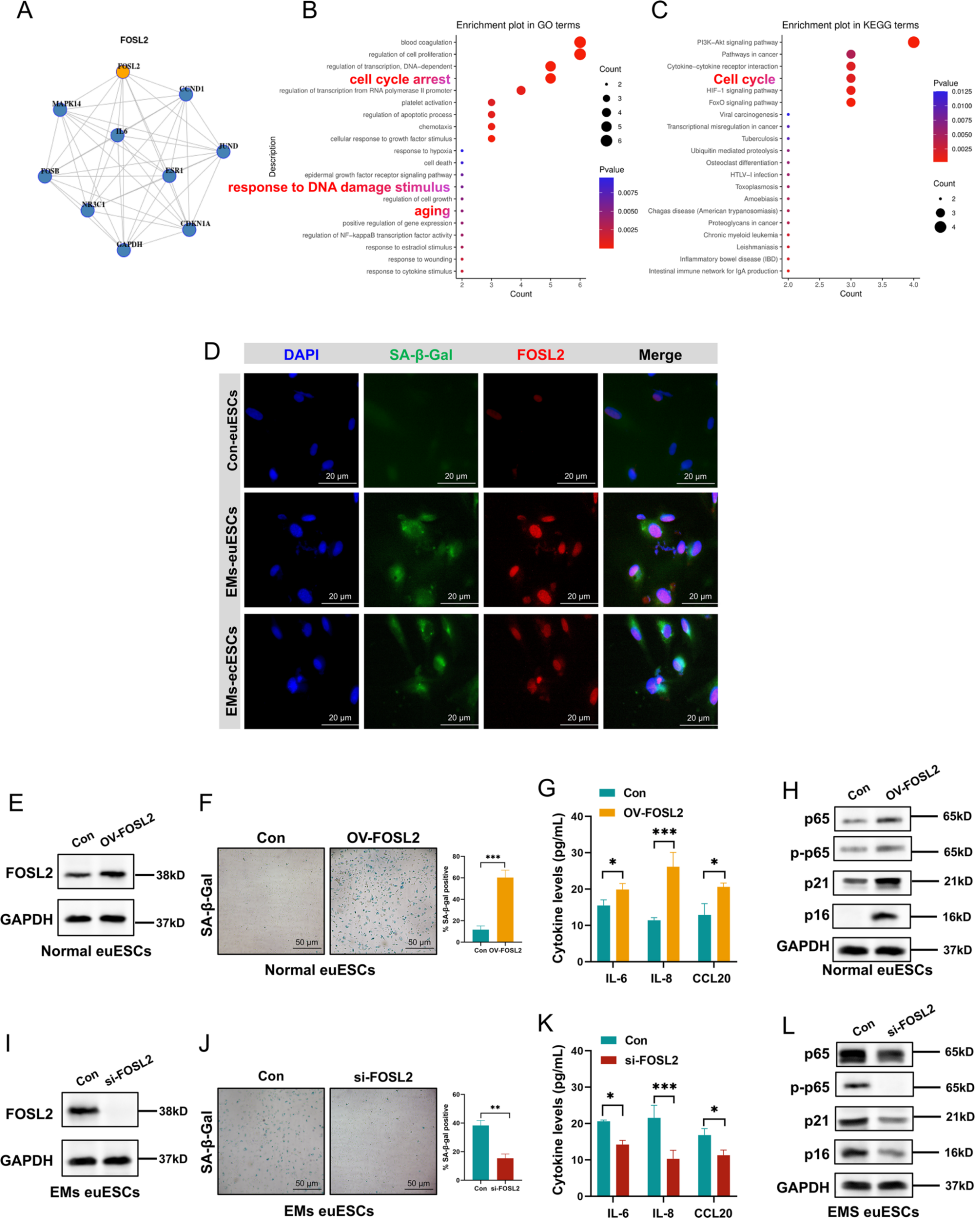
The influence of FOSL2 on the SASP of ESCs. **A** Potential target gene regulated by FOSL2 predicted by STING. **B** GO enrichment analysis and **C** KEGG enrichment analysis for the potential target genes regulated by FOSL2. **D** Representative fluorescent images of FOSL2 (red fluorescence) and SA-β-Gal (green fluorescence) in Con-euESCs, EMs-euESCs and EMs-ecESCs (scale bar, 20 μm). Quantification of FOSL2-positive, SA-β-Gal-positive, and double-positive cells is shown in the right panel. The influence of (**E**) FOSL2 overexpression on (**F**) SA-β-Gal level (scale bar, 50 μm), (**G**) SASP factors secretion, (**H**) p21, p16 and NF-κB pathway protein expression in normal euESCs. The influence of (**I**) FOSL2 knockdown on (**J**) SA-β-Gal level (scale bar, 50 μm), (**K**) SASP factors secretion, (**L**) p21, p16 and NF-κB pathway protein expression in EMs euESCs. * *P* < 0.05, ** *P* < 0.01, *** *P* < 0.001.

We further predicted the target genes of FOSL2 and identified IL6 and CDKN1A as direct targets of this TF (Fig. 4A). Enrichment analysis of potential target genes regulated by FOSL2 revealed its involvement in regulating cell proliferation, cell cycle arrest, aging, and the NF-κB signaling pathway (Fig. 4B, C). Additionally, the nuclei of SA-β-Gal-positive cells in EMs-euESCs and EMs-ecESCs exhibited positive expression of FOSL2 (Fig. 4D). Quantitative analysis confirmed that the percentages of FOSL2-positive cells, SA-β-Gal-positive cells, and, most importantly, cells co-expressing both markers were significantly increased in EMs-euESCs and EMs-ecESCs compared to the control group (Fig. 4D). These results highlight the potential role of FOSL2 in the pathobiology of EMs, possibly influencing SASP of ESCs.

To further explore the role of FOSL2, we overexpressed it in ESCs from non-EMs controls and observed a significant increase in the proportion of SA-β-Gal-positive cells (Fig. 4E, F). FOSL2-overexpressed ESCs also secreted higher levels of IL6, IL8, and CCL20 compared to control ESCs (Fig. 4G). Western blot revealed that FOSL2 overexpression in ESCs led to upregulation of p21, p16, and activation of the NF-κB pathway (Fig. 4H). Conversely, knockdown of FOSL2 in EMs-euESCs resulted in a decrease in SA-β-Gal staining, less secretion of IL6, IL8, and CCL20, and reduced protein expression of p21, p16, and phosphorylated p65 (Fig. 4I–L). In summary, our findings suggest that FOSL2 regulates cellular senescence and SASP through NF-κB pathway activation in ESCs of EMs.

### The SASP factors secreted by ESCs induce the recruitment and polarization of macrophages

The successful implantation of ectopic ESCs within the peritoneal cavity requires their ability to evade immune surveillance. It has been demonstrated that the elevated presence of M2 macrophages in the peritoneal cavity of EMs patients contributes to the persistence of ectopic ESCs^23^. Notably, the number of M2 macrophages increases progressively from stage I to stage IV of EMs, instead of M1 macrophages^24^. Previous studies have shown that IL8 and CCL20 regulated macrophage recruitment and drive M2 macrophage polarization, leading to the immunosuppressive microenvironment^25,26^. Thus, we investigated whether SASP-associated ESCs in EMs, which secrete these cytokines, could induce macrophage recruitment and M2 polarization.

**Fig. 5:**
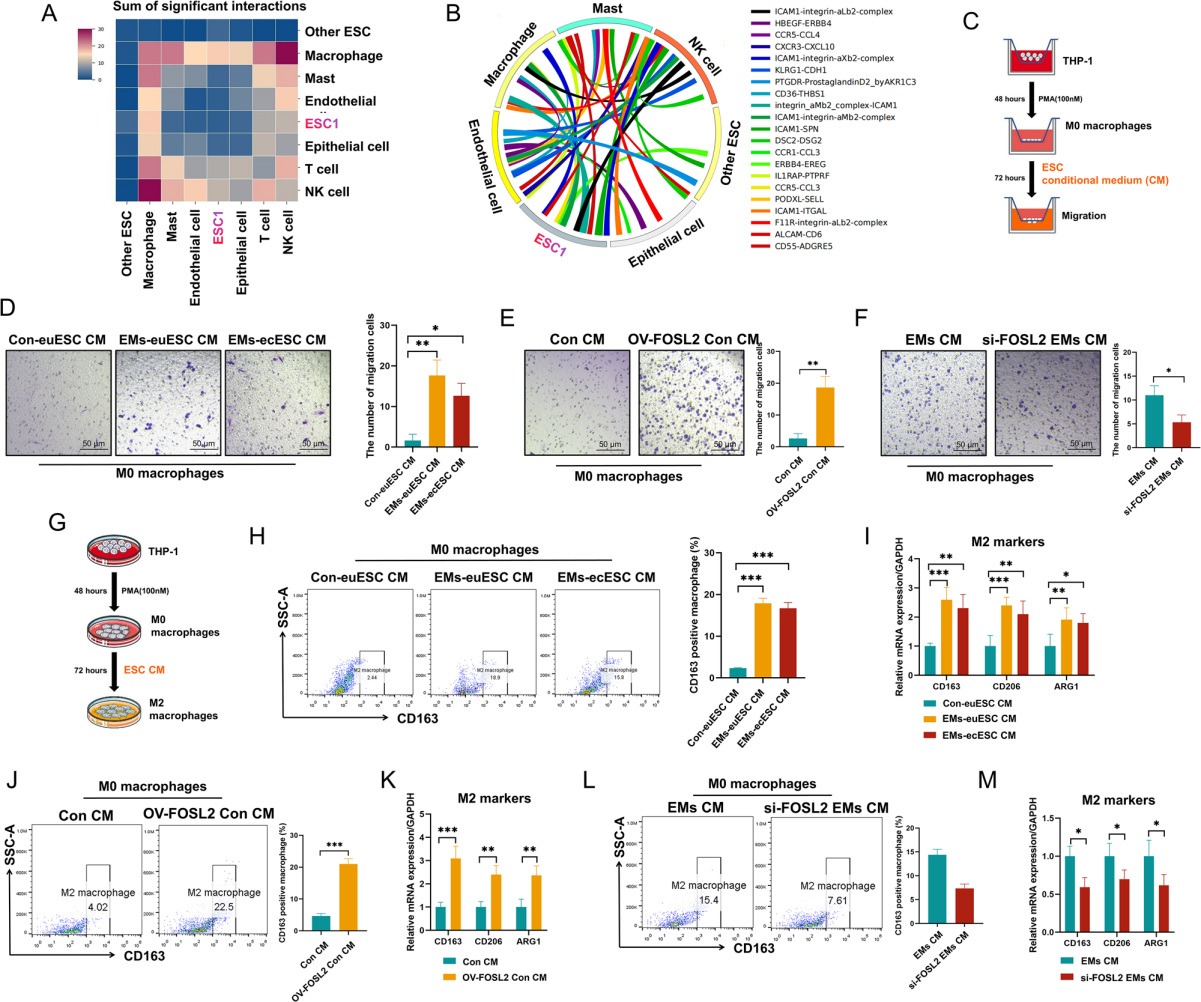
Effects of FOSL2-regulated SASP-associated ESCs on macrophages. **A** Heatmap showing intercellular communication among the cell groups of the scRNA-seq. **B** Chord diagram illustrating the key ligand-receptor pairs among different cell types. **C** The schematic diagram for the migration experiment of M0 macrophages (derived from THP-1) in vitro. **D** The impacts of conditional media derived from Con-euESCs, EMs-euESCs and EMs-ecESCs on M0 macrophage migration (Scale bar, 50 µm). **E** The impact of CM from FOSL2-overexpressed ESCs on M0 macrophage migration (Scale bar, 50 µm). **F** The impact of CM from si-FOSL2 ESCs on M0 macrophage migration (Scale bar, 50 µm). **G** A schematic diagram for M2 polarization of M0 macrophages (derived from THP-1) in vitro. **H** The M2-phenotype macrophage ratio and **I** mRNA levels of M2 markers (CD206, CD163 and ARG1) in M0 macrophages (derived from THP-1) exposed to CM derived from Con-euESCs, EMs-euESCs and EMs-ecESCs. **J** The impact of CM from FOSL2-overexpressed ESCs on M2 polarization (Scale bar, 50 µm). **K** The impact of CM from FOSL2-overexpressed ESCs on mRNA levels of M2 markers. **L** The impact of CM from si-FOSL2 ESCs on M2 polarization (Scale bar, 50 µm). **M** The impact of CM from si-FOSL2 ESCs on mRNA levels of M2 markers. * *P* < 0.05, ** *P* < 0.01, *** *P* < 0.001.

We first performed intercellular communication analysis on the scRNA-seq data. The results demonstrated robust communication between the previously identified ESC1 subcluster (the SASP-secreting ESC subgroup) and macrophages (Fig. 5A). The key ligand-receptor pairs involved in these pathways are displayed in Fig. 5B. To further clarify the effects of ESC1 on macrophage function, we treated THP-1 cells with phorbol 12-myristate 13-acetate (PMA) to induce M0 macrophage differentiation. These M0 macrophages were subsequently exposed to ESC-conditional medium (CM), and their migratory capabilities were evaluated (Fig. 5C). M0 macrophages treated with CM from EMs-euESCs and EMs-ecESCs exhibited significantly higher migratory activity compared to those treated with CM from Con-euESCs (Fig. 5D). Since FOSL2 regulates SASP in EMs-ESCs, we next investigated the effects of FOSL2 overexpression and knockdown in ESCs on macrophage migration. As shown in Fig. 5E, F, CM derived from FOSL2-overexpressed ESCs promoted M0 (Supplementary Fig. S4) macrophage migration, while knockdown of FOSL2 in EMs-euESCs reduced macrophage migration.

We further employed flow cytometry to investigate the effects of ESC-conditioned medium (CM) on macrophage polarization (Fig. 5G), which revealed that M0 macrophages cultured with CM from EMs-euESCs and EMs-ecESCs expressed higher levels of the M2 macrophage marker CD163 (Fig. 5H). Consistently, CM from EMs-euESCs and EMs-ecESCs also increased mRNA expression of M2 macrophage markers CD163, CD206, and ARG1 (Fig. 5I). We also examined the expression of the M2 marker IL10 and the M1 marker CD86. This analysis confirmed a significant upregulation of IL10 in macrophages treated with CM from EMs-euESCs and EMs-ecESCs, while CD86 expression remained unchanged, supporting a specific induction of M2 polarization (Supplementary Fig. 3A).

We further investigated the effects of FOSL2 overexpression and knockdown in ESCs on M2 polarization. As shown in Fig. 5J-M, CM derived from FOSL2-overexpressed ESCs increased the frequency of CD163-positive macrophages, and elevated the mRNA levels of M2 macrophage markers. This was further substantiated by the upregulation of IL10 and a modest decrease in CD86 expression in macrophages treated with CM from FOSL2-overexpressing ESCs (Supplementary Fig. 3B). In contrast, knockdown of FOSL2 in EMs-euESCs reduced their abilities to induce M2 polarization, highlighting the critical role of FOSL2 in the immunomodulatory landscape of EMs. This was again confirmed by the attenuated IL10 induction following treatment with CM from FOSL2-knockdown ESCs (Supplementary Fig. 3C). Collectively, these findings imply that the secretory profile of EMs ESCs actively contributes to macrophage recruitment and M2 polarization, and dysregulated overexpression of FOSL2 in EMs ESCs plays a critical role in shaping the immunosuppressive environment mediated by M2 macrophages within EMs.

### FOSL2 is regulated by PGE2/cAMP/PKA signaling pathway in ESCs

Ample research has consistently demonstrated elevated levels of prostaglandin E2 (PGE2) in the eutopic endometrium and lesions of EMs patients^27^. It has also been shown that PGE2 modulates gene expression in ESCs through the activation of the cAMP/PKA signaling pathway^28^. Additionally, PGE2 has been observed to rapidly induce the expression of FOSL2 mRNA and protein in osteoblasts^29^. Based on these findings, we aimed to investigate whether FOSL2 expression could be regulated by the PGE2/cAMP/PKA signaling pathway in ESCs.

We first confirmed that levels of PGE2 and its downstream messenger cAMP were significantly elevated in EMs ESCs (Supplementary Fig. 4A, B) and that PGE2 induced cAMP production (Supplementary Fig. 4C). Crucially, quantitative Western blot analysis revealed that PGE2 treatment significantly increased FOSL2 protein levels in normal ESCs (Supplementary Fig. 4D). More importantly, we found that pharmacological inhibition of cAMP with SQ22536, or inhibition of PKA with H89, both led to a significant reduction in the elevated FOSL2 levels in EMs-euESCs and EMs-ecESCs (Supplementary Fig. 4E, F). Taken together, our data suggest that the PGE2/cAMP/PKA signaling pathway regulates FOSL2 expression in EMs ESCs.

## Discussion

In this study, we identified a unique subgroup of ESCs characterized by the secretion of pro-inflammatory SASP in both the eutopic endometrium and lesions of EMs patients. This ESC subgroup secretes a range of pro-inflammatory factors, particularly IL8 and CCL20, which promote macrophage recruitment and M2 polarization. This process likely plays a crucial role in the establishment of the peritoneal immunosuppressive environment in EMs patients, thereby impairing the clearance of ESCs and facilitating lesion development.

Disruption of the pelvic immune environment, particularly involving macrophages, is a critical factor in the development of EMs. Research has shown that macrophages in EMs patients exhibit an increased pro-inflammatory profile and reduced phagocytic capacity compared to those in healthy individuals^30^. Furthermore, both endometriotic lesions and PF from EMs patients exhibit an elevated presence of M2 macrophages^31^. M2 macrophages have been implicated in the impaired clearance of retrograde endometrial debris, as well as in angiogenesis and fibrosis in EMs^32,33^. However, the specific mechanisms driving the dysregulation of M2 macrophages in EMs remain poorly understood.

Senescent cells, through the secretion of SASP factors, can induce M2 macrophage polarization. Although cellular senescence is traditionally considered a permanent cell cycle arrest, which suppresses the growth of ectopic ESC, recent perspectives recognize that senescence is a reversible process involving dynamic epigenetic and transcriptional remodeling^10^. This process may be exploited by cells to resist stress-induced death and evade immune surveillance through M2 macrophage polarization. For example, Mazzoni et al. demonstrated that senescent thyroid tumor cells can initiate M2 macrophage polarization via upregulation of COX-2, promoting tumor survival^34^. Other SASP factors, such as M-CSF^35^, CCL20^36^, IL6, and IL8^37^, have also been shown to mediate M2 polarization, reshaping the immune microenvironment. Similar phenomena have been observed in the pathogenesis of type 1 diabetes and liver fibrosis^38,39^. This study is the first to demonstrate that senescent ESCs can induce M2 macrophage polarization via the secretion of IL8 and CCL20, highlighting a novel mechanism in the pathophysiology of EMs. Despite the intrinsic slow growth of senescent ESCs, the peritoneal immunosuppressive environment they help create may support the survival of surrounding non-senescent ESCs, suggesting that senescent cells contribute to a pro-EMs microenvironment.

Our study also identified that FOSL2, a member of the AP-1 superfamily, drives the SASP in EMs-ESCs. FOSL2 has been reported to play a key role in cellular aging through inflammatory responses and is a major barrier to human somatic cell reprogramming^40–42^. Previous research has shown that FOSL2 directly regulates the transcription of key SASP-related genes, including RELB (a subunit of NF-κB)^43,44^, MMP9^45^, IL6^17^, and CCL28^15^, while indirectly modulating IL-1β transcription^46^. Our study revealed that FOSL2 orchestrates the SASP in EMs-ESCs by regulating the expression of IL6, IL8, and CCL20. We observed pronounced nuclear expression of FOSL2 in senescent EMs-ESCs, and the overexpression of FOSL2 induced senescence and SASP in ESCs, while FOSL2 knockdown had the opposite effect. These results underscore the pivotal role of FOSL2 in modulating SASP in ESCs, suggesting its regulatory function in the pathogenesis of EMs. A significant limitation of our study was the lack of specific cellular markers for identifying and isolating senescent ESCs, which hindered focused investigations into their characteristics and behavior.

In addition to regulating SASP, we found that FOSL2 may also modulate the forkhead box O (FOXO) signaling pathway (Fig. 4C), which controls the transcription of decidualization markers, such as prolactin and IGFBP1^47^. This suggests that abnormal FOSL2 expression might also play a role in EMs-related infertility. Taken together, these findings highlight the potential therapeutic efficacy of targeting FOSL2 to reverse SASP of ESCs in EMs.

The inflammatory factor PGE2, which is elevated in the PF, serum, and lesions of EMs patients, likely modulates FOSL2 expression via the cAMP/PKA signaling pathway. Both cAMP and PKA stimulation enhanced FOSL2 expression in ESCs, while inhibiting cAMP or PKA activity reduced FOSL2 levels. Notably, previous studies have shown that PGE2 induces cellular senescence in fibroblasts, hepatocytes, and other cell types^48,49^. In EMs, PGE2 may promote cellular senescence through the activation of FOSL2 and SASP-associated genes. Additionally, senescent cells thEMselves secrete PGE2^34^, a phenomenon we observed in EMs-euESCs and EMs-ecESCs, where PGE2 secretion was increased. This suggests the possibility of a PGE2–FOSL2–SASP–PGE2 feedback loop in ESCs of EMs. Such a loop could perpetuate chronic inflammation in the pelvic cavity and eutopic endometrium, contributing to lesion development, fibrosis, and impaired endometrial receptivity.

In summary, our study demonstrates the existence of an ESC subgroup characterized by pro-inflammatory SASP in EMs, which promotes macrophage recruitment and M2-phenotype polarization. Furthermore, we identify the transcription factor FOSL2 as a key factor associated with this SASP-positive state. Certainly, further experiments are needed to confirm this hypothesis. These findings enhance our understanding of the “retrograde menstruation phenomenon” theory of EMs and further illustrate the causes of chronic pelvic inflammation in EMs patients. Thereby, targeting FOSL2 to counteract SASP could represent a promising therapeutic approach for EMs.

## Methods

### Ethics approval and consent to participate

The research was approved by the Ethics Committee of Zhongda Hospital Affiliated to Southeast University in accordance with the Declaration of Helsinki. All enrolled patients provided their informed consent, which included agreement to the collection of tissue samples and clinical information without any impact on the pathological diagnosis process.

### Clinical sample preparation

Ectopic endometriosis lesions, eutopic endometrium and peritoneal fluid samples were obtained from patients with pathologically confirmed EMs. Peritoneal fluid and eutopic endometrium were also collected from control individuals without evidence of EMs.

### Primary endometrial stromal cell isolation and culture

Primary eutopic ESCs (euESCs) were isolated from the endometrium of EMs patients and non-EMs controls. Primary ectopic ESCs (ecESCs) were isolated from EM lesions. Isolation of ESCs was performed as described previously^50^. The tissue samples were washed 3 times with phosphate-buffered saline (PBS) and minced into small fragments of approximately 1 mm^3^ and subjected to enzymatic digestion at 37 °C for 1 h using a mixture of 2 mg/mL collagenase type IV, 0.2 mg/mL DNase I, and 0.2 mg/mL protease (all from Sigma-Aldrich, USA). The cell suspension was passed through 100 μm and 40 μm cell strainers to filter out debris and epithelial cells, respectively. Then ESCs were cultured in DMEM/F12 medium (Gibco, USA) with 10% fetal bovine serum (FBS, Gibco, USA), 0.1 mg/mL streptomycin and 100 U/mL penicillin (Gibco, USA) at 37 °C in a humidified atmosphere containing 5% CO_2_. After overnight culture, the medium containing blood cells and debris was carefully removed and replaced with fresh medium. The purity of the cultured ESCs was verified by immunofluorescent staining using vimentin, a specific marker for stromal cells. THP-1 cells were cultured in RPMI 1640 medium (Gibco, USA) containing 10% FBS at 37°C in a humidified atmosphere containing 5% CO_2_.

### Pharmacological treatment of cells

For signaling pathway investigation, normal euESCs were treated with PGE2 (20 μM, MCE, USA) for 24 h. To assess the role of the cAMP/PKA pathway, EMs-euESCs were cultured for 24 h in the presence of either the adenylyl cyclase inhibitor SQ22536 (100 μM, MCE, USA) or the PKA inhibitor H89 (5 μM, MCE, USA). Control cells were treated with an equivalent volume of DMSO. Following treatment, cells were harvested for protein extraction and Western blot analysis.

### Data acquisition

The single-cell RNA-seq (scRNA-seq) data of endometrium samples (GSE214411) were obtained from the Gene Expression Omnibus (GEO, http://www.ncbi.nlm.nih.gov/geo/) database.

### scRNA-seq analysis

The scRNA-seq data were processed using the scanpy Python package. Cells expressing fewer than 200 genes and mitochondrial content >10% of total transcripts were excluded. Post quality control and normalization, principle component analysis (PCA) was performed on the scaled variable gene matrix, and the top 10 principle components were used for clustering and dimensional reduction. Cell clusters were visualized by using Uniform Manifold Approximation and Projection (UMAP).

### Cell type annotation

The cell type identity of each cluster was determined with the expression of canonical markers found in the DEGs using SynEcoSys database. Heatmaps/dot plots displaying the expression of markers used to identify each cell type were generated by the scanpy Python package.

### Functional enrichment analysis and cluster scoring

Using the scanpy Python package, we associated these gene sets with our single-cell RNA-seq data to identify functional enrichment across different cell types or conditions. AUCell quantified the degree of gene set enrichment by calculating the area under the curve of the enrichment score for each gene set in every cell. To investigate the potential functions of DEGs, the Gene Ontology (GO) and Kyoto Encyclopedia of Genes and Genomes (KEGG) analysis were used. Pathways with *P*_adj_ < 0.05 were considered significantly enriched. Gene Ontology gene sets, including molecular function (MF), biological process (BP), and cellular component (CC) categories, were used as reference. AUCell quantified the degree of gene set enrichment by calculating the area under the curve of the enrichment score for each gene set in every cell.

### Pseudotime trajectory analysis

Pseudotime trajectory construction was performed using the pyVIA Python package. We employed the UMAP method for dimensionality reduction and visualized the pseudotime trajectory using the “plot_cells” function.

### Total RNA extraction, reverse transcription and quantitative real-time PCR

Total RNA was extracted from cells using TRIzol reagent (Takara, Japan). RNA quantification and purification were carried out at a 260/280 ratio on a NanoDrop One/One^c^ spectrophotometer (Thermo Scientific, USA). 1 μg of total RNA was reverse transcribed in a 20 μL volume by HiScript IV RT SuperMix for qPCR (Vazyme, China). Real-time PCR was performed with Taq Pro Universal SYBR qPCR Master Mix (Vazyme, China). Specific primers used for PCR amplification were synthesized with the sequences as shown in Table 1. The relative expression levels of target genes were analyzed using the 2^−ΔΔCt^ method.

**Table 1:** Primers used for real-time PCR

Genes	Forword primer (5’-3’)	Reverse primer (5’-3’)
FOSL2	GTCACTCCGGGCACCTCGAAC	TTGGTCCCCGCTGCTACTGCT
ATF3	CTGGAAAGTGTGAATGCTGAAC	ATTCTGAGCCCGGACAATAC
NFκB1	AGAGGATTTGCTGAGGGTTG	TGCTGAGGATTCTGTCGTGT
REL	CAACCGAACATACCCTTCTATCC	TCTGCTTCATAGTAGCCGTCT
CD163	TTTGTCAACTTGAGTCCCTTCAC	TCCCGCTACACTTGTTTTCAC
CD206	GCCAAATGACGAATTGTGGA	CACGAAGCCATTTGGTAAACG
ARG1	GTGGAAACTTGCATGGACAAC	AATCCTGGCACATCGGGAATC

### Protein extraction and western blot analysis

Cells were washed three times in ice-cold PBS ahead of being lysed in radio-immunoprecipitation assay (RIPA) lysis buffer (Beyotime, China) supplemented with phenylmethylsulfonyl fluoride (PMSF, Servicebio, China) and protease inhibitors cocktail (Servicebio, China) on ice for 10 min and then centrifuged at 12,000 rpm for 15 min at 4 °C. BCA Protein Assay Kit (Servicebio, China) was used to quantify the protein concentrations. Extracted proteins were uniformly mixed with sodium dodecyl sulfate–polyacrylamide gel electrophoresis (SDS–PAGE) sample loading buffer (Servicebio, China) and boiled for 10 min at 95 °C. 20 μg protein was loaded and separated by 10-12% SDS–PAGE, transfered to 0.45 μm polyvinylidene fluoride (PVDF) membranes (Millipore, Germany). Membranes were blocked in 5% nonfat milk diluted by Tris-buffered saline containing 0.05% Tween 20 (TBST) for 1 h at room temperature and incubated with primary rabbit antibody against FOSL2 (CST, 19967, 1:1000, USA), p16 (Proteintech, 10883-1-AP, 1:2000, China), p21 (Proteintech, 10355-1-AP, 1:2000, China), p65 (Proteintech, 10745-1-AP, 1:2000, China), p-p65 (Proteintech, 28842-1-AP, 1:1000, China) and GAPDH (Proteintech, 10494-1-AP, 1:10000, China) at 4 °C overnight. The next day, membranes were washed three times with TBST for 15 min and incubated with secondary antibodies (Proteintech, China) at room temperature for 1 h. The membranes were washed again, and blots were visualized by enhanced chemiluminescence (ECL, Vazyme, China).

### Cell viability measurements

ESCs were seeded in 96-well plates overnight. The working solution containing 10 µL Cell Counting Kit-8 (CCK-8, Beyotime, China) and 90 μL DMEM/F12 was added to each well. The plates were incubated at 37 °C for 1 h and then measured at an absorbance of 450 nm by using a multimode microplate reader (Thermo Scientific, USA). Cell viability (CV, %) was analyzed with the following Eq.(1).

1 $${\rm{CV}}( \% )=[({{\rm{A}}}_{{\rm{sample}}}{\rm{\mbox{-}}}{{\rm{A}}}_{{\rm{blank}}})/({{\rm{A}}}_{{\rm{standard}}}{\rm{\mbox{-}}}{{\rm{A}}}_{{\rm{blank}}})]\times 100.$$

### Cell transfection for gene knockdown and overexpress

Small interfering RNAs (siRNA) explicitly targeting FOSL2 (si-FOSL2) and negative control (si-NC) were obtained from Hanbio (China). The FOSL2 (ov-FOSL2) and negative control (ov-NC) were constructed and designed on the overexpression plasmid and synthesized by Hanbio (China). ESCs were seeded in 6-well plates at about 80% confluent density. According to the manufacturer’s protocol, we prepared an siRNA lipid complex containing 250 µL opti-MEM (Gibco, USA), 7.5 µL Lipofectamine™ 3000 (Invitrogen, USA) and 50 nM siRNA. For plasmid transfection, the complex contains 250 µL opti-MEM, 5 µg plasmid, 7.5 µL Lipofectamine™ 3000 and 10 µL P3000^TM^. The transfection mixture was replaced 24 h later with DMEM/F12 with 10% FBS.

### Immunofluorescence

ESCs were fixed in 4% paraformaldehyde for 15 min, washed in PBS three times, and stained with CellEvent™ Senescence Green dye (Thermo Scientific, USA) at 37 °C for 2 h. Then these cells were immersed in Triton X-100 for 10 min. Subsequently, ESCs were incubated overnight with rabbit primary antibodies against Vim (Proteintech, 10366-1-AP, 1:200, China) or FOSL2 (CST, 19967, 1:100, USA). The slides were then washed in PBS with 0.5% Tween, and both secondary antibodies Alexa Fluor^®^ 594 (1:500, Goat Anti-Rabbit IgG H&L, Proteintech, China) were added. A qualitative analysis was performed using a Zeiss Fluorescence microscope (Oberkochen, Germany).

### Immunohistochemistry

Tissue samples (4 μm thick) were prepared and mounted on slides. Staining was performed using primary antibodies against p21 (Proteintech, 10355-1-AP, 1:400, China), IL6 (Abcam, ab233706, 1:200, UK), FOSL2 (CST, 19967, 1:200, USA). Immunohistochemistry was performed using a BenchMark ULTRA IHC/ISH Marking Platform (Ventana Medical SystEMs, USA). The UltraView Universal DAB Detection Kit (Ventana Medical SystEMs, USA) was used to detect the marking signal. At the end of each stage, the platform underwent successive washes with Tris-based buffer solution. An ultra-coverslip protection solution was used for permanent preservation. Photomicrography was performed with a VS200 OLYMPUS microscope (Olympus, Japan).

### Enzyme-linked immunosorbent assay (ELISA)

Cell culture supernatant and peritoneal fluid (PF) from EMs patients and the control group were used to measure IL6, IL8 and CCL20 concentration by ELISA kit (Proteintech, China). After all steps of ELISA, including adding samples, incubating, washing and coloring, were completed under the manufacturer’s instructions, the absorbance at 450 nm was detected within 15 min. The concentration of cytokines was calculated based on a standard curve.

### Transwell migration assay

Cell migration was performed in Corning transwell insert chambers (8.0 μm pore size) according to the manufacturer’s instructions. THP-1 cells were cultured in the upper well transwell insert chamber with medium (500 μL) containing 100 nM PMA for 48 h to induce M0 macrophage differentiation. Then replace the culture medium in the lower chamber with ESC-conditional medium (CM), incubating for another 72 h. Then the transwell insert chamber membrane was fixed with 4% paraformaldehyde for 15 min and stained with crystal violet for 15 min. Finally, images of cells were observed and randomly taken under a fluorescence microscope. Every 6 fields were counted for each sample.

### Senescence-associated β-galactosidase (SA-β-Gal) staining

SA-β-Gal staining was performed at pH 5.5 for tissues and pH 6.0 for ESCs with Senescence β-Galactosidase Staining Kit (Beyotime, China) as previously described^51^. Briefly, frozen endometrium and endometriosis tissue sections or adherent cells were fixed with 0.5% glutaraldehyde in PBS for 15 min, washed with PBS supplemented with 1 mM MgCl_2_ and stained at 37 °C overnight in PBS containing 1 mM MgCl_2_, 1 mg/mL X-Gal and 5 mM potassium ferricyanide and potassium ferrocyanide. The slides were subsequently rinsed in PBS and mounted for determination. Every 6 representative images of each sample were quantified.

### Flow cytometry analysis

Flow cytometry was used to determine the CD163 expression on Macrophages. Macrophages were harvested and centrifuged at 400 × *g* for 5 min, incubated with phycoerythrin-conjugated monoclonal antibody specific for human CD163 (eBioscience, 12-1639-42, USA) for 30 min at 4 °C. Data acquisition was done in Attune™ NxT Flow cytometer (Thermo Scientific, USA) and analysis with Flowjo software (BD Biosciences, USA).

### Statistical analysis

We performed three independent experiments. All data were collected in a computerized database and analyzed with the software SPSS version 19.0 (IBM, USA). Unless otherwise indicated, data of quantitative variables were presented as means ± SEM. For data variables with normal distribution and independent-samples Student’s t-test was used for two groups, while one-way ANOVA followed by Bonferroni’s posttests was used for multiple group comparison. The test used to determine significance across groups in each experiment can be found in the figure legends. A value of *P* < 0.05 was considered as statistically significant.

## Supplementary information

Supplementary information

## Data Availability

The single-cell RNA sequencing data re-analyzed in this study are publicly available in the Gene Expression Omnibus (GEO) database under accession number GSE214411. All other datasets generated and/or analyzed during the current study are not publicly available due to ethical restrictions and the protection of patient privacy, but are available from the corresponding author on reasonable request.
