# Supplementary material for: FOSL2-driven SASP in endometrial stroma promotes the inflammation of endometriosis
Source: NPJ Aging. 2026 Jul 24;12(1):101. doi: 10.1038/s41514-026-00447-w (PMC13400612; doi:10.1038/s41514-026-00447-w)
Supplement: Supplementary file 1 — Supplementary information [file 41514_2026_447_MOESM1_ESM.pptx]

## Slide 1
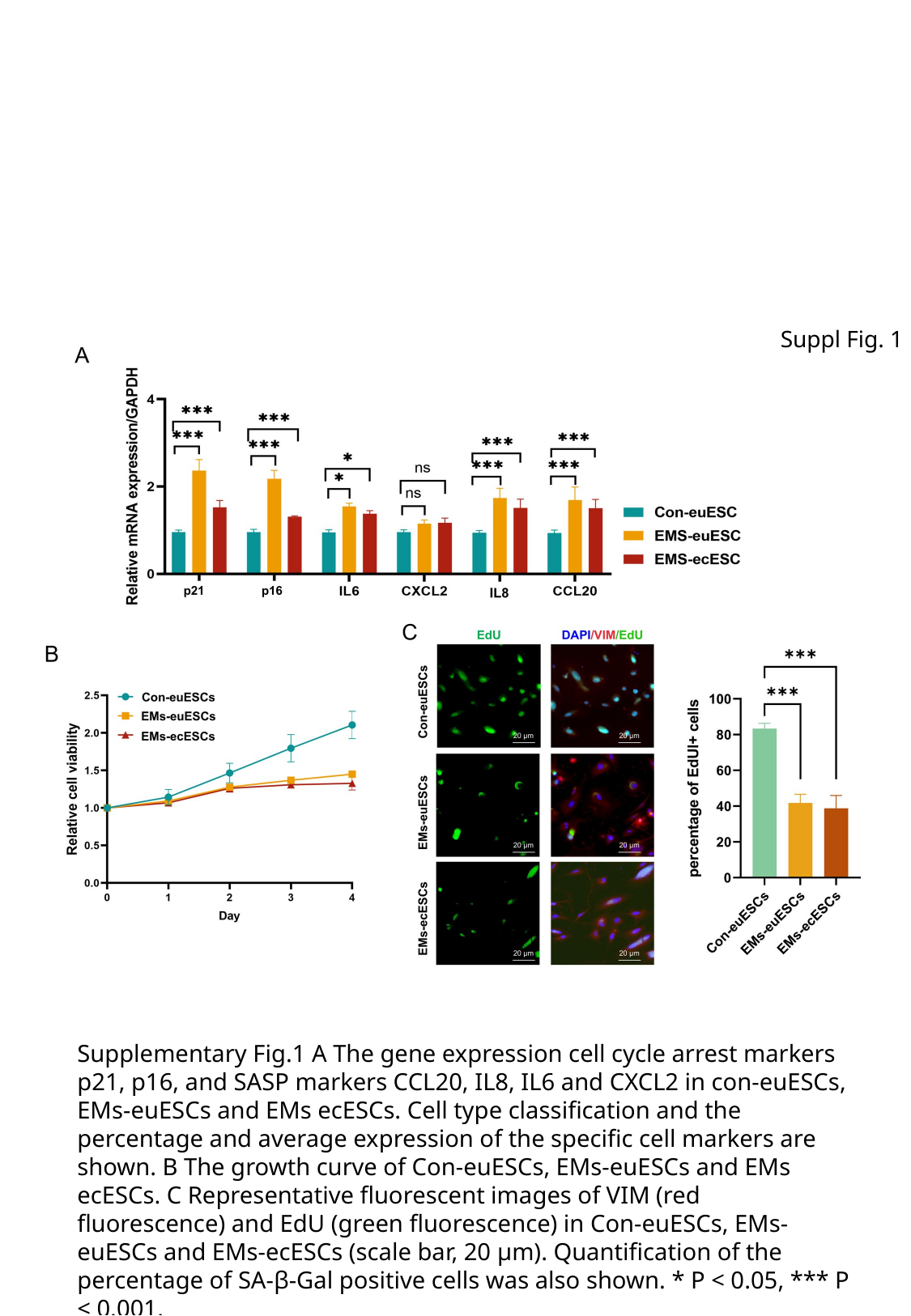

Suppl Fig. 1
Supplementary Fig.1 A The gene expression cell cycle arrest markers p21, p16, and SASP markers CCL20, IL8, IL6 and CXCL2 in con-euESCs, EMs-euESCs and EMs ecESCs. Cell type classification and the percentage and average expression of the specific cell markers are shown. B The growth curve of Con-euESCs, EMs-euESCs and EMs ecESCs. C Representative fluorescent images of VIM (red fluorescence) and EdU (green fluorescence) in Con-euESCs, EMs-euESCs and EMs-ecESCs (scale bar, 20 μm). Quantification of the percentage of SA-β-Gal positive cells was also shown. * P < 0.05, *** P < 0.001.

## Slide 2
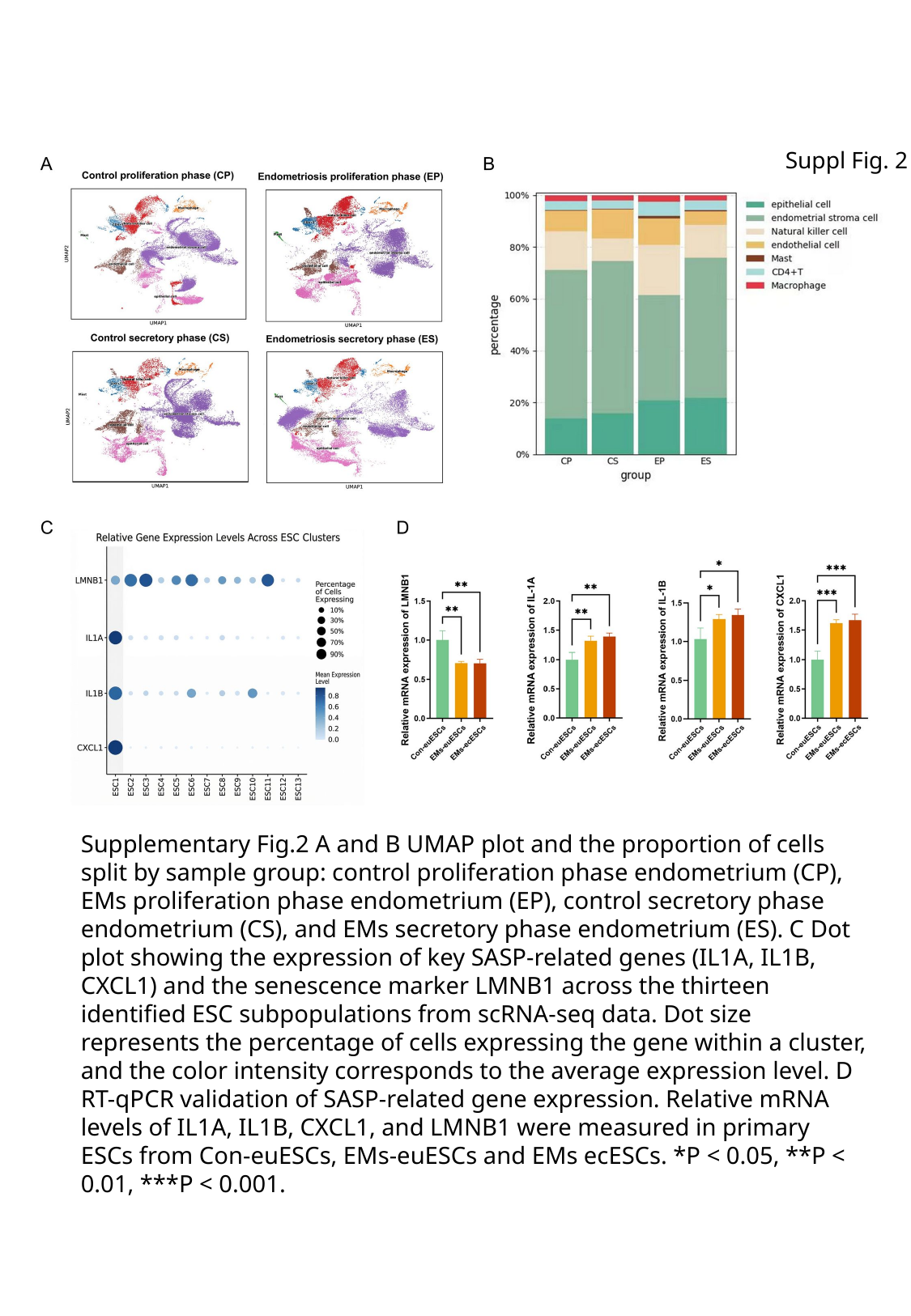

Suppl Fig. 2
Supplementary Fig.2 A and B UMAP plot and the proportion of cells split by sample group: control proliferation phase endometrium (CP), EMs proliferation phase endometrium (EP), control secretory phase endometrium (CS), and EMs secretory phase endometrium (ES). C Dot plot showing the expression of key SASP-related genes (IL1A, IL1B, CXCL1) and the senescence marker LMNB1 across the thirteen identified ESC subpopulations from scRNA-seq data. Dot size represents the percentage of cells expressing the gene within a cluster, and the color intensity corresponds to the average expression level. D RT-qPCR validation of SASP-related gene expression. Relative mRNA levels of IL1A, IL1B, CXCL1, and LMNB1 were measured in primary ESCs from Con-euESCs, EMs-euESCs and EMs ecESCs. *P < 0.05, **P < 0.01, ***P < 0.001.

## Slide 3
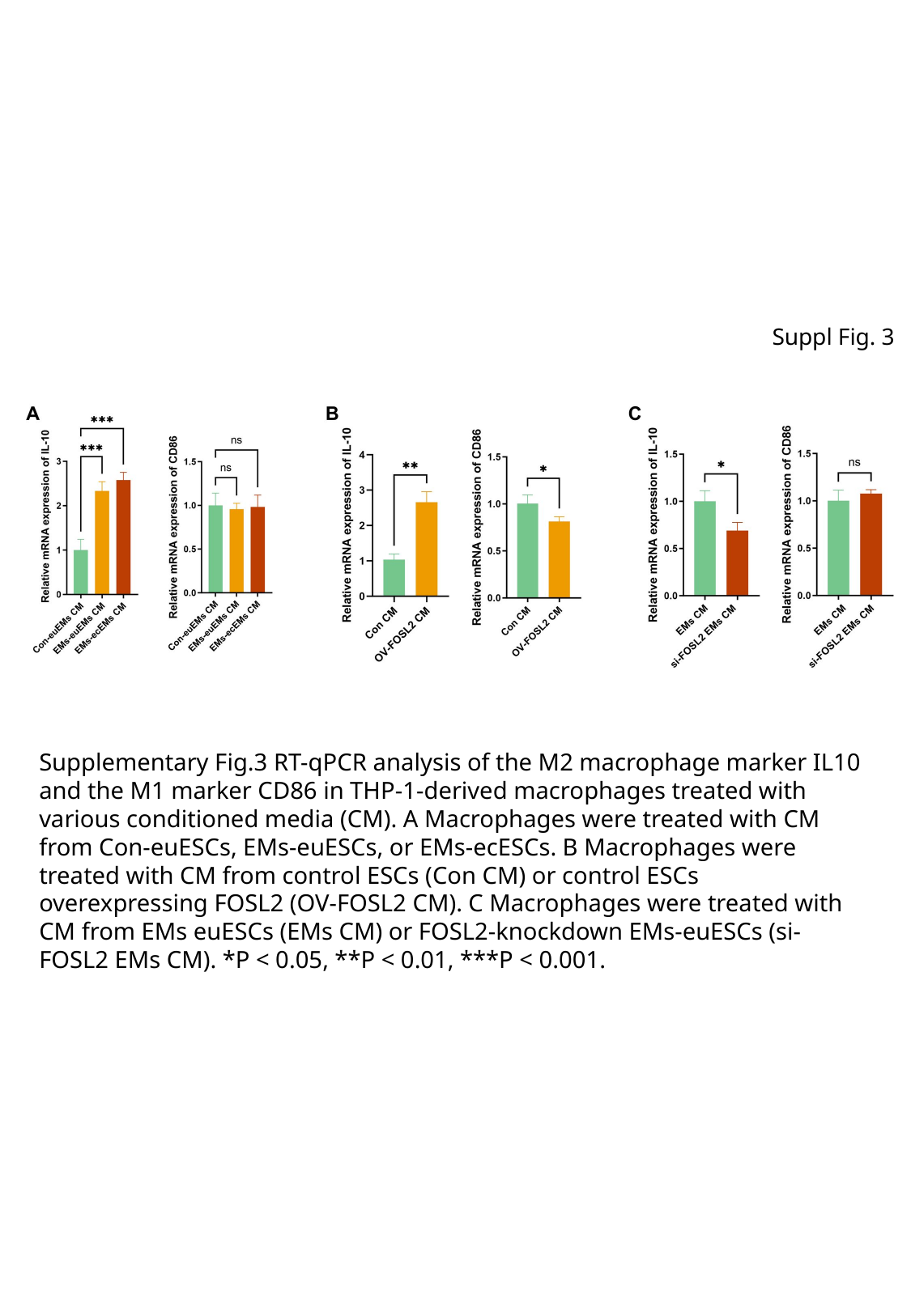

Suppl Fig. 3
Supplementary Fig.3 RT-qPCR analysis of the M2 macrophage marker IL10 and the M1 marker CD86 in THP-1-derived macrophages treated with various conditioned media (CM). A Macrophages were treated with CM from Con-euESCs, EMs-euESCs, or EMs-ecESCs. B Macrophages were treated with CM from control ESCs (Con CM) or control ESCs overexpressing FOSL2 (OV-FOSL2 CM). C Macrophages were treated with CM from EMs euESCs (EMs CM) or FOSL2-knockdown EMs-euESCs (si-FOSL2 EMs CM). *P < 0.05, **P < 0.01, ***P < 0.001.

## Slide 4
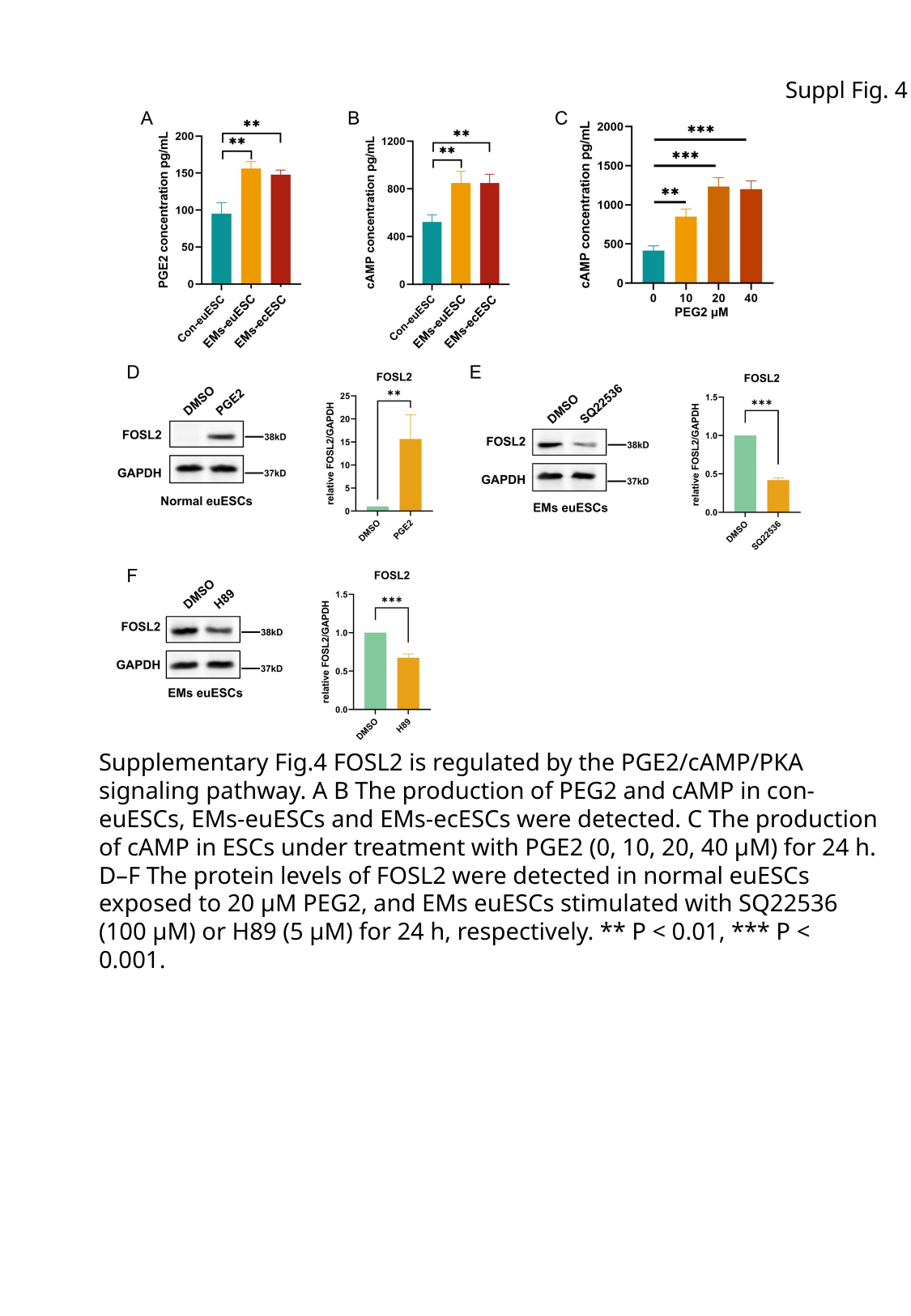

Suppl Fig. 4
Supplementary Fig.4 FOSL2 is regulated by the PGE2/cAMP/PKA signaling pathway. A B The production of PEG2 and cAMP in con-euESCs, EMs-euESCs and EMs-ecESCs were detected. C The production of cAMP in ESCs under treatment with PGE2 (0, 10, 20, 40 μM) for 24 h. D–F The protein levels of FOSL2 were detected in normal euESCs exposed to 20 μM PEG2, and EMs euESCs stimulated with SQ22536 (100 μM) or H89 (5 μM) for 24 h, respectively. ** P < 0.01, *** P < 0.001.
